# Validation of the Traditional Chinese version of Mothers on Respect Index (MORi) and Mother’s Autonomy in Decision Making (MADM) Scale

**DOI:** 10.1016/j.ijnss.2026.06.005

**Published:** 2026-06-17

**Authors:** Junyan Li, Nitya Nagesh, Heidi SL. Fan, Daniel YT. Fong, Kris Yuet Wan Lok

**Affiliations:** a School of Nursing, the University of Hong Kong, Hong Kong, China; b Monash Centre for Health Research and Implementation, Sub-Faculty of Clinical and Molecular Medicine, Monash University, Clayton, Australia; c College of Nursing, Rady Faculty of Health Sciences, University of Manitoba, Winnipeg, Manitoba, Canada

**Keywords:** Autonomy, China, Decision making, Mothers, Psychometrics, Respect

## Abstract

**Objective:**

This study aimed to translate and culturally adapt the Mothers on Respect Index (MORi) and the Mothers’ Autonomy in Decision Making (MADM) scale into Chinese, and to assess their reliability and validity among postpartum women in Hong Kong, China.

**Methods:**

The MORi and MADM scales were translated into Chinese following a rigorous forward–backward translation procedure. Between December 2023 and February 2024, Chinese-speaking women who had given birth in Hong Kong within the past five years were recruited through social media platforms. The instruments’ validity was examined through content validity, construct validity, convergent validity, discriminant validity, and known-groups validity. Reliability was assessed using internal consistency.

**Results:**

A total of 1,395 mothers participated. The Mothers on Respect Index-Revised (MORi-R) and MADM showed perfect content validity (S-CVI = 1.00). Confirmatory factor analysis identified a revised three-factor model MORi-R with satisfactory fit indices (CFI = 0.953; TLI = 0.94; SRMR = 0.07; RMSEA = 0.08). The MORi-R consisted of 13 items after Item 4 was removed. The MADM scale, comprising seven items within a single factor, remained unchanged and demonstrated good model fit (CFI = 0.995; TLI = 0.99; SRMR = 0.01; RMSEA = 0.05). The Cronbach's α was 0.86 for the MORi-R and 0.91 for the MADM scale. The MORi-R and MADM scores were moderate and showed a positive correlation (*r* = 0.57).

**Conclusions:**

The MORi-R and MADM scales were used to assess perceived disrespectful care experiences and decision-making autonomy among postpartum women in Hong Kong, China. Both instruments demonstrated desirable psychometric properties, with satisfactory reliability and validity, and may serve as valuable tools to inform clinical nursing practice and improve the quality of maternity care.

## What is known?

• The Mothers on Respect Index-Revised (MORi-R) and Mothers’ Autonomy in Decision Making (MADM) scales have been validated in diverse communities. They are effective for use in populations with limited healthcare resources.

## What is new?

• We developed traditional Chinese versions of the MORi-R and MADM scales, which demonstrated high validity and reliability in a Chinese population.
• Postpartum women in Hong Kong, China, experienced moderate levels of respectful care and decision-making autonomy, with median MORi-R and MADM scores of 61 and 29, respectively.

## 1 Introduction

A move towards a more person-centered care model is a core component of global health care reform, in which respectful maternity care and women’s autonomy are essential to high-quality care [1,2]. According to the WHO [3], respectful maternity care encompasses the provision and organization of care that uphold women’s dignity, privacy, and confidentiality, protect them from harm and abuse, and ensure informed choices and continuous support during childbirth.

However, women in both affluent and low-resource countries have encountered a disturbing range and extent of disrespect in maternity care [4]. As research advances, respectful maternity care is increasingly acknowledged as a human rights issue and is considered crucial for enhancing childbirth and delivery experiences [3]. Indeed, these disrespectful behaviors have been linked to various adverse outcomes, including prenatal anxiety [5], postpartum depression [6], and posttraumatic stress disorder [7,8]. More concerning is that fears of disrespectful maternity care may deter some women from seeking professional assistance, potentially leading to a rise in births attended by non-professionals and an increase in maternal mortality and morbidity [[9], [10], [11]]. Ensuring respectful and humane care is thus essential not only for enhancing individual care experiences but also for mitigating maternal health risks.

Patient-led decision-making is especially significant during pregnancy and childbirth [12]. A systematic review of 137 studies identified three key factors influencing women’s satisfaction with childbirth: decision-making involvement, the quality of the patient-provider relationship, and the level of caregiver support [13]. Shared decision-making is considered fundamental to patient-centered care and is closely linked to better health outcomes [14]. Another systematic review found that shared decision-making was significantly positively correlated with 54 % of emotional-cognitive outcomes, 37 % of behavioral outcomes, and 25 % of health outcomes [15].

The WHO has called for addressing evidence gaps in disrespectful care and developing tools to evaluate the quality and safety impacts of obstetric human rights violations [4]. In response, Vedam et al. developed two scales: the Mother’s on Respect Index (MORi) and the Mother’s Autonomy in Decision Making (MADM) scale [12,16]. MORi and MADM are the only validated patient-led quantitative scales for assessing respect and autonomy in maternity care. These scales have been validated across multiple cultural contexts and have demonstrated effectiveness in diverse populations [9,12,[16], [17], [18], [19], [20]]. The MORi currently has validated versions in Canada, the United States, Germany, and Australia, as well as in Dutch [4,8,9,17,18]. The MADM scale has been validated for use in the United Kingdom, Greece, Australia, the Netherlands, and Turkey [4,[17], [18], [19], [20]]. Although MORi and MADM focus on different conceptual aspects of maternity care, they can be combined to assess its overall quality. Consequently, both scales have been translated and validated in Australian and Dutch to facilitate their use in future research [17,18]. While most research focuses on resource-limited areas [21], where disrespect is more evident, respectful maternity care remains a “blind spot” in resource-rich countries [22].

Although instruments have been developed in Hong Kong, China to assess childbirth experience [23], a specific tool to facilitate discussions on respect and informed choice between providers and maternity patients is currently lacking. When developing tools to measure disrespect and abuse during childbirth, Freedman et al. [23] emphasized the necessity of defining these concepts according to local cultural and care contexts. In response to this, this study aimed to develop traditional Chinese versions of the MORi and MADM scales and evaluate their psychometric properties (reliability and validity) in a Chinese population. A further objective was to assess the level of respect and decision-making autonomy experienced by women in Hong Kong’s maternity care system, thereby enhancing the understanding of local maternity care quality.

## 2 Methods

### 2.1 Study design

A cross-sectional survey was conducted in Hong Kong, China, from December 2023 to February 2024. The study adopted an analytical design, with reporting guided by EQUATOR standards and instrument validation conducted in accordance with COSMIN guidelines [24]. Initially, both instruments were translated into Traditional Chinese to ensure semantic and idiomatic equivalence. Subsequently, the translated scales underwent cultural and conceptual adaptation. Finally, their applicability for assessing maternal experiences was evaluated.

### 2.2 Ethical approval

Approval of the study was granted by the Institutional Review Board of The University of Hong Kong/Hospital Authority Hong Kong West Cluster on 7th Nov 2023 (Ref No: UW22-690). Informed consent was obtained before survey completion. Participation was anonymous, and confidentiality was ensured. Respondents were free to withdraw from the study at any stage.

### 2.3 Translation procedure

Permission and authorization for translating the original questionnaire into traditional Chinese were obtained from the original authors. The MORi and MADM were translated into Traditional Chinese using a forward-backward translation process in accordance with WHO guidelines for translating and adapting instruments [25], following a multistep approach [26]. The English versions of the MORi and MADM scales were forward-translated into Traditional Chinese by two bilingual translators, both native Chinese speakers and health professionals. Two additional native Chinese-speaking bilingual translators, who were blinded to the original instruments, performed the back-translation into English. A native English speaker then compared the back-translation with the original English version. Two bilingual experts with backgrounds in maternal care and cultural adaptation of health assessment tools reviewed the translated versions, ensuring their cultural and linguistic relevance to the target population. During the expert review, it was noted that Item 4 might exhibit conceptual overlap with Item 5 in MORi. Still, due to a lack of statistical evidence at that stage, both items were retained. The final Traditional Chinese versions of the MORi and MADM scales were subsequently prepared.

### 2.4 Scale evaluation and validation procedure

#### 2.4.1 Study settings

Two research assistants disseminated a one-page recruitment information sheet via Facebook and approximately 20 WhatsApp discussion groups for mothers. These platforms were selected due to their widespread use among the target population, enabling effective outreach and engagement with mothers in Hong Kong, China. Interested participants then contacted the research assistant to obtain the online survey link. Survey responses were collected via the Qualtrics software system. To minimize the likelihood of multiple responses from a single participant, Qualtrics was configured to permit only one response per IP address.

#### 2.4.2 Participants

Eligible participants were women aged 18 or older who had been pregnant or given birth in Hong Kong within the past 5 years of data collection and were able to complete the Traditional Chinese versions of the MORi and MADM scales. This cross-sectional survey, part of a larger study, yielded a larger sample. Although the overall sample size was calculated based on the primary outcome of the main study, the target sample size for this specific study outcome was 210 women. This target aligns with the recommendation of a sample size ten times the number of items in the health measurement tool being evaluated [27]. As the MORi (14 items) and MADM (7 items) scales comprise a total of 21 items, 210 completed questionnaires were required. Based on an anticipated 61 % response rate, estimated from a German study [8], approximately 344 women were recruited to achieve the target sample size.

#### 2.4.3 Measurements

The self-administered survey, available in Traditional Chinese, includes sociodemographic and maternal characteristics (e.g., age, education, maternal health problems) and the target validation tools (MORi and MADM scales).

The 14-item MORi measured respectful care, the nature of the patient-provider relationship, and person-centered care [12]. The MORi, when originally developed, demonstrated good internal consistency. Cronbach's α was 0.85 in the Canadian sample and 0.94 in the U.S. sample. Nevertheless, no validity indices were provided in the initial validation studies [12].

The 7-item MADM scale [16] evaluated the decision-making process during maternity care. The original MADM scale demonstrated excellent reliability, with Cronbach’s α exceeding 0.90 across all samples. Its construct validity and one-dimensionality were supported by factor analysis, which yielded a single factor with strong loadings (0.74–0.95) and a clear 90-degree angle in the scree plot [16].

The MORi and MADM items were presented on a 6-point Likert scale, ranging from 1 (completely disagree) to 6 (completely agree). The traditional Chinese MORi comprises 14 items, with a higher total score indicating greater experiences of respectful maternity care [12]. Items 4 and 8–14 were reverse-scored. The traditional Chinese MADM scale consists of 7 items, yielding a total score ranging from 7 to 42, with higher scores indicating a greater sense of autonomy [16].

In line with the original MORi scale, participants were asked to identify their primary maternity care provider during pregnancy. For consistency in the Hong Kong context, the MADM scale was adapted to include this specification (i.e., doctor, midwife, or both), which was not present in the original version.

#### 2.4.4 Data analyses

Baseline descriptive statistics were reported for all participants who completed the MORi and MADM scales at baseline, presented as frequencies and proportions. The agreement percentage for each item of both scales was also calculated. The six-point Likert responses were dichotomized, with scores 1–3 categorized as “disagreement” and 4–6 as “agreement”.

Regarding validity assessment, we examined content validity, construct validity, convergent validity, discriminant validity, and known-group validity. Content validity was determined using the Content Validity Index (CVI), which evaluates the extent to which a scale adequately reflects the construct it is intended to measure [28]. Each item was rated by experts on a 4-point scale: 4 = highly appropriate, 3 = requires minor modification, 2 = requires major modification, and 1 = not appropriate [29]. The item-level CVI (I-CVI) was calculated as the proportion of experts assigning a rating of 3 or 4 to an item, while the scale-level CVI (S-CVI) was obtained by averaging all I-CVI values [30]. Thresholds of S-CVI >0.90 and I-CVI >0.78 were considered indicative of adequate content validity [31]. The expert panel comprised two gynecologists, two nursing specialists, and one psychologist.

To assess construct validity, we performed both exploratory factor analysis (EFA) and confirmatory factor analysis (CFA) for the MORi and MADM scales. EFA was used to identify the underlying factors accounting for the shared variance across items. In contrast, CFA was used to test the extent to which the theoretically proposed models align with the observed latent structures [32]. The data were randomly split into a training set (*n* = 718) for EFA and a validation set (*n* = 677) for CFA. The EFA, using principal axis factoring with promax rotation, was conducted to identify the underlying factor structure of the scales. The number of factors to retain was determined based on eigenvalues greater than 1 and the scree plot.

Bartlett’s test of sphericity was used to test the assumption of sphericity, and the Kaiser-Meyer-Olkin (KMO) measure was used to verify the suitability of the data for factor analysis. The CFA was then performed on the validation set to evaluate the EFA-identified factor structure. For both EFA and CFA, items were assigned to the factor on which they had the highest factor loading, provided the loading was ≥0.4. Items with loadings below this threshold were considered for removal, as they may not adequately represent the intended construct [33]. Model fit for the CFA was assessed using multiple indices: the chi-square statistics (*χ*^2^), the Comparative Fit Index (CFI), the Tucker-Lewis Index (TLI), the Standardized Root Mean Square Residual (SRMR), and the Root Mean Square Error of Approximation (RMSEA) [34]. A good model fit was defined by CFI and TLI values ≥ 0.95, and SRMR and RMSEA values ≤ 0.08 [35].

Convergent validity, which reflects the degree to which theoretically related constructs are truly correlated, was assessed by calculating the Average Variance Extracted (AVE) based on standardized coefficients from the CFA. An AVE value greater than 0.50 was considered indicative of acceptable convergent validity [36].

Discriminant validity, on the other hand, examines whether theoretically distinct constructs are indeed unrelated. This is typically supported when the squared correlation (SC) between any pair of constructs is smaller than the AVE of each construct [37].

Known-group validity was assessed to evaluate the scales’ ability to differentiate between groups. Independent-samples *t*-tests were conducted to examine differences in the three factor scores and total scores across groups defined by the presence of maternal health problems, postpartum complications, and birth interventions (e.g., induction of labor), as hypothesized based on previous studies [38].

Internal consistency reliability was assessed using Corrected item-total correlations, Composite Reliability (CR), Cronbach’s α, and Cronbach's α if an item was deleted. Reliability coefficients closer to 1.00 are considered more desirable. Specifically, a Cronbach’s α below 0.40 indicates poor reliability, 0.40–0.59 questionable reliability, 0.60–0.79 acceptable reliability, and values between 0.80 and 1.00 excellent reliability [39]. Corrected item-to-total correlations were calculated to assess whether individual items correlate highly with the sum of the items. Items with high correlation coefficients indicate strong conceptual overlap, whereas items with low coefficients may not measure the same construct. Items with a correlation coefficient ≥0.2 were retained in the scale [40].

CR was calculated based on the factor loadings from CFA. Information about how much each item affected the reliability of the respective scale was provided by comparing the Cronbach’s α coefficient when the item was deleted with the overall Cronbach’s α coefficient. Furthermore, Spearman's rank correlation was used to assess the relationship between the MADM and MORi scale scores. Except for the CFA, which was conducted with AMOS 28.0, all other statistical analyses were performed with SPSS Statistics 25.0. The level of significance was set at *P* < 0.05.

## 3 Results

### 3.1 Sample characteristics

A total of 1,535 individuals responded to the survey. Invalid responses were identified based on completion times of less than 1 min, incomplete responses to study-relevant items, or duplicate entries, to ensure data quality. After screening, 1,395 valid responses were retained for analysis. The demographic characteristics are presented in Table 1.

**Table 1:** Descriptive characteristics of the study population (*n* = 1,395).

Variables	*n* (%)
Maternal age (years)	
< 25	12 (0.9)
25–36	1,030 (73.8)
> 36	353 (25.3)
Years living in Hong Kong, China	
Less than 5 years	11 (0.8)
More than 5 years	208 (14.9)
Since birth	1,176 (84.3)
Marital status	
Married	1,339 (96.0)
Not married but living with a partner	38 (2.7)
Single/separated	18 (1.3)
Education level	
Secondary school or below	428 (30.7)
College or above	967 (69.3)
Family monthly income (HKD)	
< 20,000	73 (5.3)
20,000–30,000	163 (11.7)
30,001–40,000	165 (11.8)
> 40,000	994 (71.3)
Delivery mode	
Spontaneous vaginal delivery	753 (54.0)
Assisted vaginal delivery	124 (8.9)
Planned cesarean delivery	263 (18.9)
Emergency cesarean delivery	255 (18.3)
Maternal health problem	
No	1,024 (73.4)
Yes	371 (26.6)
Labour Induction	
No	726 (52.0)
Yes	669 (48.0)
Postpartum complications	
No	1,239 (88.8)
Yes	156 (11.2)

Most participants were aged 25–36 years, and 84.3 % identified as local Hong Kong residents. Additionally, 71.3 % reported a monthly income above 40,000 HKD, suggesting that women from lower-income backgrounds and non-local residents were underrepresented in the sample. Nearly half of the women underwent labor induction during their first childbirth, 26.6 % experienced health problems during pregnancy, and 11.2 % reported postpartum complications.

### 3.2 Validity

#### 3.2.1 Content validity

The back-translated version of MADM demonstrated overall semantic equivalence with the terminology used in the original scale. In addition, the content validity of the MORi and MADM was confirmed through a panel of five experts. Except for Item 4, the MORi items demonstrated excellent content validity, with I-CVI values of 1.00. In contrast, Item 4 had an I-CVI of 0.00, suggesting limited content relevance and representativeness. The S-CVI was 0.93 when all 14 items of the MORi were considered and increased to 1.00 after Item 4 was removed. The panel indicated that the MADM demonstrated high relevance and representativeness, as reflected in the content validity indices (S-CVI = 1.00, I-CVI = 1.00).

#### 3.2.2 Construct validity

Bartlett’s test of sphericity for the two scales was significant (*P* < 0.001), and the KMO measure of sampling adequacy was 0.88 for MORi and 0.89 for MADM, both of which support the use of factor analysis for these data. For MORi, the EFA results indicated three factors with eigenvalues greater than 1, accounting for 66.5 % of the cumulative variance. Except for Item 4, which had a factor loading less than 0.4, all other items had loadings above 0.6. Therefore, all items were retained for the time being (Appendix A). With the inclusion of all 14 items from the original MORi in the CFA, Item 4 had a standardized coefficient of 0.33, indicating a weak relationship with Factor 1. Given that experts raised concerns about the inclusion of Item 4 during the translation and expert review phase, it was ultimately removed due to its weak statistical association and limited applicability in this specific cultural context. The resulting 13 items formed the Mothers on Respect Index-Revised (MORi-R). The resulting three factors consist of 3 to 6 items: “sense of autonomy and comfort” (6 items), “perceptions of discrimination” (3 items), and “modified behavior” (4 items). The factor structure of MORi-R is illustrated in Appendix B. For MADM, the EFA results indicated a single factor with an eigenvalue greater than 1, accounting for 66.1 % of the cumulative variance. Each item exhibited a factor loading above 0.5, supporting its retention in the scale. Detailed item loadings are provided in Appendix C. The resulting factor structure of the MADM is presented in Appendix D. CFA indicated that the MORi-R factor model showed an acceptable fit to the data, with a chi-square value of 285.39, *df* = 59, *P* < 0.001, CFI = 0.953, TLI = 0.94, SRMR = 0.07, RMSEA = 0.08, and a 90 %*CI* of 0.07–0.08. The MADM factor model showed a good fit, with a chi-square value of 25.112, *df* = 9, *P* = 0.003, CFI = 0.995, TLI = 0.99, SRMR = 0.01, RMSEA = 0.05, and a 90 %*CI* of 0.03–0.08.

#### 3.2.3 Convergent validity

The MORi-R demonstrated adequate convergent validity across its three factors, with AVE values ranging from 0.57 to 0.74, including sense of autonomy and comfort (AVE = 0.57), perceptions of discrimination (AVE = 0.62), and modified behavior (AVE = 0.74). The MADM scale also showed satisfactory convergent validity (AVE = 0.59). All values exceeded the recommended threshold of 0.50, indicating acceptable convergent validity.

#### 3.2.4 Discriminant validity

The calculated Pearson correlation between the MORi-R and MADM was 0.57 (*P* < 0.001), indicating a moderate positive relationship between the two scales.

The Pearson correlations between the three factors of the MORi-R were 0.54 (Factor 1 and Factor 2), 0.17 (Factor 1 and Factor 3), and 0.35 (Factor 2 and Factor 3) (*P* < 0.001). The corresponding SCs were 0.29, 0.03, and 0.12, respectively. These SC values were all lower than the AVE of the individual factors, indicating that each factor shared more variance with its own items than with other factors. Therefore, the three factors of the MORi-R demonstrated satisfactory discriminant validity. As the MADM is a unidimensional scale, discriminant validity was not evaluated.

#### 3.2.5 Known-group validity

Three groups were selected based on key maternal health factors: maternal health problems, labor induction, and postpartum complications, as these factors are expected to influence experiences of disrespect and decision-making autonomy in maternity care. To assess known-groups validity, independent t-tests were conducted to compare the mean scores of the MORi-R and MADM scales across these groups. The *t*-test results indicated that both the MORi-R and MADM scales demonstrated good known-groups validity. Specifically, mothers with maternal health problems reported lower scores for perceived respect (*t* = 2.16, *P* = 0.03) and autonomy in decision making (*t* = 3.65, *P* < 0.001). Similarly, significantly lower scores were reported by mothers who experienced postpartum complications (*t* = 3.57, *P* < 0.001); Autonomy (*t* = 3.56, *P* < 0.001), and those who underwent labor induction (Respect: *t* = 1.98, *P* = 0.048; Autonomy: *t* = 2.36, *P* = 0.018). All group differences were statistically significant (Appendix E).

### 3.3 Reliability

#### 3.3.1 Corrected item-total correlations

All items showed a corrected item-total correlation above 0.3 for the MORi-R and above 0.5 for the MADM, indicating moderate to good item reliability (Appendix A and Appendix C).

#### 3.3.2 Cronbach’s α coefficient and composite reliability

The Cronbach’s α values, if any item were deleted, consistently remained above 0.8 for both scales. The overall Cronbach’s α for the MORi-R was 0.86, and for the MADM, it was 0.91, demonstrating good internal consistency (Appendix A and Appendix C). The CR values for the MORi-R were 0.89, 0.83, and 0.88, while the CR for the MADM was 0.91. With CR values above 0.7 for all factors across both scales, each factor demonstrated strong convergent validity and internal consistency.

### 3.4 Experience of respect and autonomy in decision making

More than half of the participants reported feeling comfortable asking questions of their care providers while making decisions (88.3 %), and that they accepted (86.8 %) or declined (72.9 %) offered care, with personal (85.6 %) or cultural preferences (94.1 %) respected. In contrast, agreement with negatively worded items was significantly lower than with positively worded items, indicating a higher perceived respect index. Only 9 % of participants felt they were treated poorly by their doctor or midwife because of race, ethnicity, cultural background, or language (Appendix A). The median MORi-R score was 61 (5th/95th percentile: 52–67, range 55).

All items had an agreement rate exceeding 60 %. Specifically, 86.5 % of participants reported that their doctor or midwife respected their choices, while 81.4 % felt that they were helped to understand all the information. Additionally, 81 % reported being able to choose the best care options they considered (Appendix C). The median MADM score was 29 (5th/95th percentile: 25–34, range 35).

## 4 Discussion

The results support the feasibility, reliability, and known-group validity of these measures for use with pregnant and postpartum women in Hong Kong, China. At the same time, the initial translated MORi model demonstrated poor model fit; a revised version (MORi-R) was developed, showing adequate validity and reliability. Both MORi-R and MADM are efficient patient-reported measures suitable for evaluating respect and autonomy in decision-making, thereby supporting person-centered maternity care.

The EFA was employed because no predefined factor structure was available to explore the scale’s latent factors within the local cultural context. EFA facilitated the identification and validation of a factor structure aligned with Hong Kong’s cultural context, thereby ensuring both the scale’s cultural relevance and psychometric validity. The results revealed that the EFA identified three dimensions, consistent with the original scale's factor structure. This suggests that, despite cultural differences, the experiences of respect and decision-making autonomy among women in Hong Kong, China, exhibit structural similarities to those observed in other regions.

This finding underscores the scale’s cultural adaptability and affirms the stability of its original factor structure across diverse cultural contexts. Furthermore, the application of EFA in this study demonstrates its efficacy in uncovering the underlying structure of a scale across various cultural settings, thereby enhancing the robustness and validity of the study’s findings.

The scales in this study exhibit strong psychometric properties. As documented in existing literature, the MORi has been validated in the original [12,16], German [8], Australian [18], and Dutch [17] versions. At the same time, the MADM is available in these versions as well as in Greek [20] and Turkish [19]. Similarly, in our study with the Hong Kong population, the MADM scale retained all 7 items and demonstrated robust psychometric properties in both EFA and CFA.

The MORi-R scale was revised by reducing the number of items from 14 to 13, following the removal of Item 4 due to its suboptimal factor loadings in both the EFA and CFA and inadequate content validity. While the original scale [12] faced similar issues but retained the item for practical reasons, our decision to exclude it was based on statistical criteria. Excluding Item 4 does not alter the scale’s core construct, which continues to focus on disrespect in maternity care. The theoretical foundation remains intact, with the adjustment improving measurement precision without deviating from the original framework. Item 4 was found to exhibit conceptual overlap with Item 5, thereby diminishing its discriminant validity in measuring the intended construct. This adjustment improved the cultural adaptability and psychometric properties of the MORi-R in Hong Kong, China, but also limits its comparability with studies using the original version.

From a dimensional perspective, the MADM scale in this study aligns with other validation studies, demonstrating a single dimension [17]. The presence of only one eigenvalue greater than 1 and high loadings on a single factor support its unidimensionality and good construct validity. In contrast, our analysis of the MORi-R scale identified three distinct factors in both EFA and CFA, which correspond closely to the three sections of the original scale.

It is noteworthy that the original MORi-R scale exhibited varying factor structures during EFA, including instances of cross-loadings [12]. Despite these issues, a single-factor structure was retained for practical reasons. The German version also included items with lower factor loadings, and similar unidimensional results have been reported in other countries [8]. Several factors may explain this discrepancy between our study and others. First, we used advanced principal component extraction methods and varimax rotation during EFA, resulting in different factor structures compared to studies that used unweighted least squares methods without rotation, which tend to preserve more of the original data characteristics. Second, our detailed CFA analysis revealed the multidimensional nature of the MORi-R scale. Additionally, differences in respondent characteristics across cultural contexts may influence factor structures, as cultural interpretations and response patterns affect how items are perceived and answered [22]. Therefore, the observed discrepancy in dimensions may stem from these methodological and cultural variations.

The median MORi-R score in this study was 61, and the median MADM score was 29, suggesting a moderate level of respect and autonomy [12,16]. Despite the MORi-R scale having one fewer item than in the original study [12], both scales yielded higher scores in the Hong Kong Chinese population compared to North American studies. In those studies, the median scores were 50 for the MORi and 25 for the MADM, which were categorized as “moderate” in terms of respect and autonomy [16].

The observed differences may stem from the insufficient representation of non-local Hong Kong, Chinese women, individuals with low socioeconomic status, or individuals with low income in our sample. Although research indicates that social media can effectively recruit participants from lower socioeconomic backgrounds [41], women from these backgrounds, as well as those from diverse ethnic groups, recent immigrants, or refugees with low literacy rates, remain underrepresented. This likely reflects the fact that the recruitment social media groups were predominantly composed of local mothers and women of higher socioeconomic status, leading to the underrepresentation of other groups [17].

In contrast, scores in this study were lower than those observed in Australia [18] and the Netherlands [17]. For example, the median MORi score was 74 in Australia and 77 in the Netherlands, both of which were categorized as “high respect.” Similarly, the median scores for the A-MADM scale were 36 in Australia and 35 in the Netherlands, indicating a “high level of autonomy” [17,18]. These studies were conducted in high-income populations, which may account for differences in cultural perceptions of respect and autonomy, healthcare practices, or healthcare systems.

The analysis of known-groups validity revealed significant insights into construct validity. Mothers with maternal health issues, postpartum complications, and those who underwent induction reported lower scores in perceived respect and autonomy in decision-making. This finding aligns with studies from Canada [12] and the Netherlands [18], where women with pregnancy complications reported lower MORi scores and reported reduced perceptions of respect. These results support previous research and suggest a decline in provider attention to aspects such as intuition, empathy, and care during unexpected medical issues and childbirth interventions [42].

Our study found a positive correlation between MORi-R and MADM scores (*r* = 0.57): participants who perceived their care as highly respectful also experienced greater autonomy in decision-making. Conversely, lower autonomy was associated with perceptions of less respectful care. This close link suggests that enhancing one aspect may positively influence the other. Future nursing practices and interventions should therefore aim to improve both respect and autonomy to offer more comprehensive, person-centered care. In Australian (*r* = 0.86) and German (*r* = 0.82) studies, the correlation between these scales was higher [8,18], potentially due to differences in cultural context, sample characteristics, or scale adaptations.

## 5 Strengths and limitations

First, this study is the first to evaluate the effectiveness of the MORi and MADM scales in capturing disrespect and mistreatment during childbirth within the Chinese obstetric context, providing novel insights into their applicability in this setting. Second, the translated and validated MORi-R and MADM scales effectively measure the maternity experiences of Chinese women, demonstrating high validity and reliability in a localized context. Finally, the study's large sample size supports the use of CFA [43], reinforcing the statistical significance and reliability of the findings. However, this study has several limitations. First, the use of convenience sampling via online platforms may have introduced self-selection bias, as mothers with particularly positive or negative experiences might have been more inclined to participate [44]. This could affect the representativeness of the sample and limit the generalizability of the findings. Second, the sample exhibited higher levels of education and family income, which may restrict the applicability of the results to other populations in Hong Kong, China. Third, a potential limitation is recall bias, as the inclusion criteria focused on women who had been pregnant or given birth in Hong Kong within the past five years. This time frame may have led to retrospective reporting of experiences related to disrespect and mistreatment during childbirth, potentially affecting the accuracy of participants’ recollections. These limitations in sample characteristics may affect the external validity of the findings. Although the sample predominantly consisted of local mothers with higher socioeconomic status, this is unlikely to affect the assessment of the scale's psychometric properties, including reliability and construct validity.

## 6 Conclusion

The traditional Chinese versions of the MORi-R and MADM scales demonstrated strong psychometric properties, establishing them as valid and reliable for assessing experiences of respect and autonomy in decision-making within Hong Kong’s maternity care context. These scales provide valuable means to evaluate provider-patient relationships and an individual's ability to lead decision-making throughout the maternity care process.

## Data availability statement

The datasets generated during and/or analyzed during the current study are available from the corresponding author upon reasonable request.

## CRediT authorship contribution statement

**Junyan Li:** Writing – original draft, Formal analysis. **Nitya Nagesh:** Data curation, Writing – review & editing. **Heidi SL. Fan:** Data curation, Writing – review & editing. **Daniel YT. Fong:** Writing – review & editing, Supervision. **Kris Yuet Wan Lok:** Conceptualization, Formal analysis, Methodology, Writing – review & editing, Supervision.

## Declaration of competing interest

The authors have declared no conflict of interest.
