# Supplementary material for: Validation of the Traditional Chinese version of Mothers on Respect Index (MORi) and Mother’s Autonomy in Decision Making (MADM) Scale
Source: Int J Nurs Sci. 2026 Jun 17;13(4):435–41. doi: 10.1016/j.ijnss.2026.06.005 (PMC13424731; doi:10.1016/j.ijnss.2026.06.005)
Supplement: Multimedia component 1 [file mmc1.docx]

**女性围产期受尊重指数及自主决策量表汉化及验证研究**

李君艳，Nitya Nagesh，樊思乐，方以德，骆月云

【**摘要**】

**目的** 汉化女性围产期受尊重指数（Mothers on Respect Index, MORi）和自主决策量表（Mothers Autonomy in Decision Making Scale, MADM），并在中国香港有分娩经历的女性中验证中文版量表的信效度。

**方法** 采用正向-反向翻译过程汉化MORi和 MADM。于2023年12月至2024年2月在中国香港通过社交媒体招募能够阅读中文、且在过去5年内在香港有分娩经历的香港籍女性。效度包括内容效度、结构效度、收敛效度、判别效度、已知组别效度验证量表的效度。采用内部一致性验证量表的信度。

**结果** 共有1 395名女性参与了研究。修订后的MORi（MORi-Revised, MORi-R）和 MADM的内容效度为1.00。验证性因子显示，中文版MORi-R包括3因子模型，模型拟合指数显示可接受拟合(CFI = 0.953; TLI = 0.94; SRMR = 0.07; RMSEA = 0.08)。MORi-R在删除第4项后，共包含13个条目。MADM由7个条目组成，属于单一因子结构，未作修改，且表现出良好的模型拟合(CFI = 0.995; TLI = 0.99; SRMR = 0.01 RMSEA = 0.05)。MORi-R的Cronbach’s α为0.86，MADM的Cronbach’s α为0.91。MORi-R和MADM得分处于中等水平，并呈正相关（*r* = 0.57）。

**结论** MORi-R 与 MADM 量表用于评估中国香港围产期女性感知到的不尊重照护体验及决策自主权水平，两项工具均展现出理想的心理测量学属性，具备良好信度与效度，能够为临床护士优化产科护理实践提供重要参考依据。

【**关键词**】自主权；中国；决策过程；母亲；心理测量学；尊重

通信作者：Kris Yuet Wan Lok, E-mail:[krislok@hku.hk](mailto:krislok@hku.hk)
