# Supplementary material for: Validation of the Traditional Chinese version of Mothers on Respect Index (MORi) and Mother’s Autonomy in Decision Making (MADM) Scale
Source: Int J Nurs Sci. 2026 Jun 17;13(4):435–41. doi: 10.1016/j.ijnss.2026.06.005 (PMC13424731; doi:10.1016/j.ijnss.2026.06.005)
Supplement: Multimedia component 2 [file mmc2.docx]

Appendix A. MORi frequency table, item – total statistics and factor structure (*n* = 1,395)

| Items | % who  agreed  with item | Factor loadings ^a,b^ | Corrected  item – total  correlation | Cronbach’s α  if item  deleted |
| --- | --- | --- | --- | --- |
| Factor 1 Overall while making decisions about my pregnancy or birth care |  |  |  |  |
| 1. I felt comfortable asking questions | 88.3 | 0.71 | 0.55 | 0.85 |
| 1. I felt comfortable declining care that was offered | 72.9 | 0.63 | 0.48 | 0.86 |
| 1. I felt comfortable accepting the options for care that my doctor or midwife recommended | 86.8 | 0.85 | 0.59 | 0.85 |
| 1. I felt pushed into accepting the options my doctor or midwife suggested* | 53.0 | 0.39 | - | - |
| 1. I chose the care options that I received | 88.0 | 0.82 | 0.59 | 0.85 |
| 1. My personal preferences were respected | 85.6 | 0.85 | 0.63 | 0.85 |
| 1. My cultural preferences were respected | 94.1 | 0.76 | 0.56 | 0.85 |
| Factor 2 During my pregnancy I held back from asking questions or discussing my concerns because |  |  |  |  |
| 1. My doctor or midwife seemed rushed* | 45.3 | 0.87 | 0.63 | 0.85 |
| 1. I wanted maternity care that differed from what my doctor or midwife recommended* | 44.2 | 0.66 | 0.55 | 0.85 |
| 1. I thought my doctor or midwife might think I was being difficult* | 38.1 | 0.85 | 0.66 | 0.84 |
| Factor 3 During my pregnancy I felt that I was treated poorly by my doctor or midwife because of |  |  |  |  |
| 1. My race, ethnicity, cultural background or language* | 9.4 | 0.82 | 0.50 | 0.86 |
| 1. My sexual orientation and/or gender identity* | 14.1 | 0.86 | 0.39 | 0.86 |
| 1. My type of health insurance or lack of insurance* | 17.3 | 0.79 | 0.37 | 0.86 |
| 1. A difference of opinion with my caregivers about the right care for myself or my baby* | 18.8 | 0.62 | 0.57 | 0.85 |

*Note*: ^a^ Extraction Method: Principal Axis Factoring. ^b^ Rotation Method: Promax with Kaiser Normalization.*Reversed scored. MORi = Mothers on Respect Index.


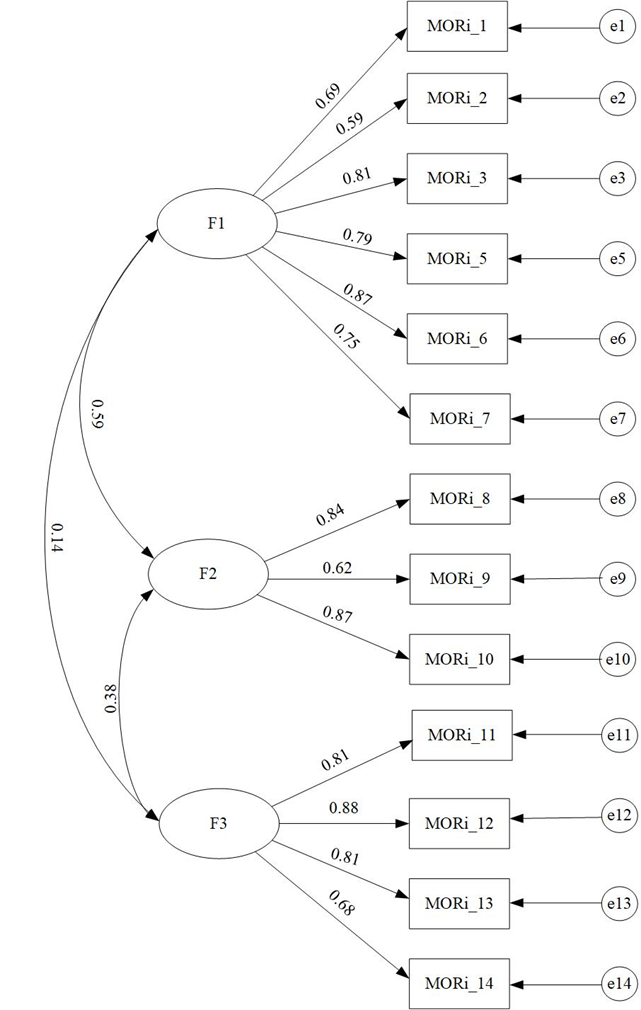


Appendix B. Factor structure of MORi-R

*Note*: MORi = Mothers on Respect Index.

Appendix C MADM frequency table, item – total statistics and factor structure (*n* = 1,395)

| Items | % who  agreed  with item | Factor loadings^a,b^ | Corrected  item – total  correlation | Cronbach’s α  if item  deleted |
| --- | --- | --- | --- | --- |
| 1. My doctor or midwife asked me how involved in decision making I wanted to be. | 65.3 | 0.58 | 0.55 | 0.92 |
| 1. My doctor or midwife told me that there are different options for my maternity care. | 74.4 | 0.79 | 0.76 | 0.90 |
| 1. My doctor or midwife explained the advantages/disadvantages of the maternity care options. | 75.2 | 0.82 | 0.77 | 0.89 |
| 1. My doctor or midwife helped me understand all the information. | 81.4 | 0.86 | 0.80 | 0.89 |
| 1. I was given enough time to thoroughly consider the different care options. | 74.1 | 0.84 | 0.78 | 0.89 |
| 1. I was able to choose what I considered to be the best care options. | 81.0 | 0.81 | 0.77 | 0.89 |
| 1. My doctor or midwife respected my choices. | 86.5 | 0.73 | 0.72 | 0.90 |

*Note*: ^a^ Extraction Method: Principal Axis Factoring. ^b^ Rotation method: Promax with Kaiser Normalization. MADM = Mothers Autonomy in Decision Making.


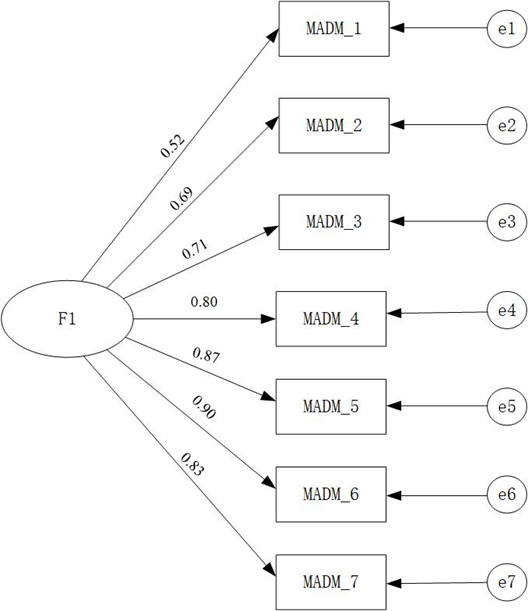


Appendix D. Factor structure of MADM

*Note*: MADM = Mothers Autonomy in Decision Making.

Appendix E Known-group comparison of Mothers on Respect Index and Mothers’ Autonomy in Decision Making Scale (*n* = 1,395)

| Variables | MORi-R | | | | MADM | | | |
| --- | --- | --- | --- | --- | --- | --- | --- | --- |
|  | *Mean* ± *SD* | *t* | *P* | *95%* *CI* | *Mean ± SD* | *t* | *P* | *95% CI* |
| Maternal health problem |  | 2.16 | 0.030 | 0.12, 2.55 |  | 3.65 | < 0.001 | 0.68, 2.24 |
| No | 59.93 ± 10.06 |  |  |  | 29.62 ± 6.42 |  |  |  |
| Yes | 58.59 ± 10.50 |  |  |  | 28.16 ± 7.02 |  |  |  |
| Labour induction |  | 3.57 | < 0.001 | 0.87, 3.01 |  | 3.56 | < 0.001 | 0.56, 1.95 |
| No | 60.51 ± 10.32 |  |  |  | 29.83 ± 6.59 |  |  |  |
| Yes | 58.57 ± 9.96 |  |  |  | 28.58 ± 6.58 |  |  |  |
| Postpartum complications |  | 1.98 | 0.048 | 0.01, 3.41 |  | 2.36 | 0.018 | 0.23, 2.43 |
| No | 59.77 ± 10.17 |  |  |  | 29.38 ± 6.57 |  |  |  |
| Yes | 58.06 ± 10.31 |  |  |  | 28.05 ± 6.89 |  |  |  |

*Note*: MORi = Mothers on Respect Index. MADM = Mothers Autonomy in Decision Making.
